# Digital Twin-Based Active Learning for Industrial Process Control and Supervision in Industry 4.0

**DOI:** 10.3390/s25072076

**Published:** 2025-03-26

**Authors:** Jessica S. Ortiz, Evelin K. Quishpe, Grace X. Sailema, Nathaly S. Guamán

**Affiliations:** 1 Departamento de Eléctrica, Electrónica y Telecomunicaciones, Universidad de las Fuerzas Armadas ESPE, Sangolquí 171103, Ecuador; 2 Departamento de Ciencias de la Energía y Mecánica, Universidad de las Fuerzas Armadas ESPE, Sangolquí 171103, Ecuador

**Keywords:** Digital Twin, virtualized environment, active learning, Industry 4.0

## Abstract

The integration of Digital Twin technology into active learning environments has been established as an innovative strategy to optimize engineering education. This study focuses on the development and evaluation of a learning tool based on Digital Twin technology, enabling interaction and communication between a physical teaching module and a virtualized environment representing the industrial cocoa production process. The system was operated by students using an S7-1200 PLC and an HMI interface developed in WinCC, facilitating real-time process monitoring and control via IoT. To assess its impact, an experimental design was implemented, comparing a pre-test using conventional teaching modules with a post-test incorporating Digital Twin technology. The results indicate a significant improvement in students’ accuracy and efficiency, with a 38% reduction in task execution time. This improvement is attributed to the system’s immersive and interactive capabilities, which allow for the simulation of actuator and sensor behavior under real-world conditions. These findings highlight the potential of Digital Twins not only as educational tools but also as valuable assets for industrial process optimization in the cocoa sector, aligning with Industry 4.0 principles.

## 1. Introduction

During the last decades, several digital tools have been developed to support both planning and management of industrial production [1,2]. These tools facilitate the collection and analysis of real-time data, enabling optimization and predictive maintenance strategies that improve industrial efficiency [3]. The advent of digital technologies has led to significant advancements in integrating intelligent, interconnected components into industrial settings. These innovations allow for remote sensing, real-time monitoring, and control of cyber–physical devices and production elements via network infrastructures, ensuring seamless integration between physical and virtual environments [4,5]. Among these advancements, the Digital Twin has emerged as a pivotal technology that bridges the gap between physical and virtual environments, allowing for real-time synchronization of industrial assets and processes [6,7]. A Digital Twin is a virtual representation of a physical system that continuously updates and evolves based on real-world data, offering opportunities for improved diagnostics, performance optimization, and scenario simulation. This capability enhances diagnostics, optimizes performance, and enables scenario simulations, making Digital Twins fundamental to Industry 4.0. They provide a foundation for intelligent automation, remote monitoring, and autonomous decision-making, ensuring early problem detection and mitigation before issues arise. Additionally, Digital Twins can dynamically respond to operational conditions, such as closing circuits when necessary or improving repetitive production steps [8,9].

While Digital Twins technology is widely utilized in industrial settings for process optimization, predictive maintenance, and operational efficiency, its application in engineering education and research is an area of growing interest [8,9]. Digital Twins are now being integrated into academic environments to support active learning methodologies, providing students with immersive and interactive experiences that closely replicate real-world industrial processes [10]. By allowing students to manipulate and analyze virtualized process variables—such as temperature, pressure, and flow rates—Digital Twins facilitate a deeper understanding of industrial automation principles and real-time decision-making dynamics [11]. In engineering education, innovative didactic tools are transforming the Active Learning Process by providing interactive and realistic environments [10]. Among these, Digital Twins stand out as one of the most advanced and promising technologies. A Digital Twin offers an accurate virtual representation of a physical system, enabling real-time interaction with its physical counterpart [11,12,13]. This model allows engineering students to immerse themselves in dynamic learning experiences, where they can analyze, experiment with, and control industrial processes without the risks inherent in real environments [4,14].

The application of Digital Twins in academic settings provides multiple benefits, such as the ability to simulate and optimize products, enhance process efficiency, and visualize system behavior under various operating conditions [15,16]. In educational contexts, this technology simplifies complex concepts by immersing students in simulated environments that faithfully replicate industrial systems [17,18]. Students can safely experiment, identify potential failures, and correct errors before they occur in real life. Despite the promising applications of Digital Twins in education, there is a need for scientific investigation into their effectiveness in bridging the gap between academic training and industrial applications. Most existing studies have focused on their pedagogical benefits, but few have addressed their role as experimental research tools for process analysis, control strategy validation, and industrial system optimization [12]. The present study aims to contribute to this knowledge gap by developing a Digital Twin framework for industrial process automation and evaluating its effectiveness in both academic and industrial contexts. This research investigates the use of Digital Twins as an industrial control tool, assessing their ability to enhance process supervision, predictive maintenance, and production efficiency through real-time data integration and Internet of Things (IoT) technologies [13,14].

A key feature of Digital Twin-based learning is the simulation of actuators, transducers, and other components of an industrial plant. Students are responsible for controlling process variables, such as temperature, pressure, or flow rate, and observing how these changes affect the overall system. In the event that a mistake is made during the execution of tasks, the system can signal faults such as fires, machinery failures, or safety issues, which generates immediate feedback and encourages a problem-solving approach [19,20]. This approach not only reinforces theoretical learning but also develops critical practical skills, such as decision-making under pressure, analyzing real-time data, and understanding the relationship between control actions and system responses. The ability to integrate simulated failures in a controlled environment provides students with an experience close to industrial reality, preparing them more effectively for the challenges they will face in their future careers. The implementation of Digital Twins in active learning environments is based on a crucial step: the complete virtualization of the industrial process [21,22]. This involves recreating key physical aspects of the work environment, such as dimensions, textures, and industry-specific operating conditions. To carry out this process effectively, advanced tools can be used such as Unity 3D (Unity Technologies, San Francisco, CA, USA), a software tool that stands out for its ability to accurately represent an industrial environment in detail [10]. Unity 3D allows the creation of interactive virtual environments in which safety standards are incorporated, realistic interaction with machinery is simulated, and avatars wearing the appropriate personal protective equipment (PPE) are used, significantly improving the immersion and realism of the simulation.

To illustrate this approach, this study focuses on the implementation of a Digital Twin for the automation of an industrial cocoa processing plant, specifically addressing classification, roasting, and peeling operations. The proposed DT integrates IoT technologies, real-time monitoring systems, and a Human–Machine Interface (HMI) for process visualization and control. The research aims to demonstrate how DTs can be used not only as an educational tool but also as a robust methodology for process analysis, optimization, and industrial automation research. Furthermore, this study contributes to the growing body of literature by providing empirical insights into the operational benefits and limitations of DT implementations in industrial environments. By adopting a dual perspective—pedagogical and industrial—this research advances the scientific understanding of Digital Twins as a transformative technology for both education and industrial automation. The findings aim to inform future developments in DT applications, supporting the ongoing evolution of intelligent manufacturing systems and workforce training in Industry 4.0.

The advancement of digital tools has given way to the use of the IoT as a key complement in the development of Digital Twins and other learning platforms [23]. In an academic environment, IoT allows students to access real-time data on the behavior of simulated industrial processes, gaining a greater understanding of system dynamics. Using sensors and actuators connected to a global network, students can monitor control variables, such as temperature, pressure, or speed, and observe how these affect overall system performance [23,24]. This gives them the opportunity to make informed decisions and adjust them in real time, fostering an interactive, hands-on approach to learning. The IoT allows students to go beyond theory and achieve a clear visualization of how the various components of an industrial system interact. They can observe the behavior of sensors and actuators in real time and make adjustments to control variables based on the data received. This dynamic interaction enhances their ability to understand the relationship between control decisions and system performance, a critical element in industrial automation [25]. By incorporating IoT into the learning environment, students also become familiar with key technologies that are transforming modern industries. In industry, IoT is increasingly being used to improve operational efficiency, optimize predictive maintenance, and facilitate remote monitoring [26,27]. By introducing these technologies into the classroom, a bridge is created between the academic and industrial environments, preparing students to meet the technological challenges of the future [26,28].

Additionally, IoT enhances student training by simulating failures and emergency scenarios based on real-time data. When integrated with Digital Twin simulations, IoT systems can detect anomalies—such as overloads or mechanical failures—enabling students to apply immediate response protocols. This not only reinforces their problem-solving skills but also fosters critical thinking, essential for their professional careers. Ultimately, incorporating IoT into Digital Twin-based learning significantly enhances the educational experience by providing a window into the real world of industrial automation and control. As technology continues to evolve, the IoT will continue to play a pivotal role in preparing engineers for the industries of the future, where connectivity and real-time, data-driven decision-making will be essential elements in process optimization and technological innovation.

In this context, the project focuses on developing a Digital Twin to automate an industrial plant dedicated to the classification, roasting and peeling of cocoa. This development seeks to integrate IoT technologies and process virtualization to improve the monitoring, manipulation and control of each stage of the production system. The implementation of a real-time supervision and data acquisition system is crucial, as it allows monitoring and controlling the operation of the plant regardless of the physical location of the supervisors, ensuring continuous and efficient control of the process. To achieve this automation, the PLC was connected to the computer where the grading and roasting process was programmed, and the data obtained were displayed on a Human–Machine Interface (HMI) designed to be user-friendly and easy to interpret. This made it easy for operators and supervisors to follow, in real time, the status of each stage of the plant. The process was initiated by a pulse on the control panel, and the correct operation of the sequence was verified by visual indicators that confirmed that the system was operating as expected.

Virtualization of the physical plant environment was done using Unity 3D, allowing operators and students to have a clear and detailed view of the layout and operation of the equipment. This level of visualization not only facilitated the understanding of the industrial process but also provided an additional layer of safety and efficiency by allowing to simulate the interaction with the plant before making changes or adjustments in the real environment. Finally, the data generated by the system were integrated with an IoT module, allowing supervisors and operators to check information in real time from multiple devices, such as smartphones or tablets, ensuring effective remote monitoring. This connectivity capability through IoT significantly improved decision-making, as managers could react proactively to any eventuality or deviation in the process, without the need to be physically present in the plant. This comprehensive approach, which combines PLC programming, visualization via HMI, and process virtualization with Unity 3D and monitoring via the IoT, represents an advanced and efficient solution for industrial automation. It also provides engineering students with an ideal platform to understand and practice real process control and monitoring, using cutting-edge technologies that are key to the digital transformation of industry.

## 2. Conceptualization

The development of a didactic tool based on Digital Twins significantly complements the learning of students in the engineering area through the manipulation, control, and supervision of virtualized actuators and sensors, the same that are found in the representation of an industrial process. That is, the virtual work environment aims to replicate the real behavior of the equipment, in order to allow students to experience and better understand complex industrial processes. The integration of Digital Twins allows students to interact with a realistic and dynamic environment, improving their understanding and skills in the control and supervision of industrial processes. In order to ensure modularity, scalability, and efficiency, the proposed Digital Twin followed a layered architecture that aligns with established Industry 4.0 frameworks.

This architecture consisted of: (a) a perception layer, in charge of capturing real-time data from sensors and actuators, as well as monitoring key variables such as temperature, pressure, and flow; (b) a processing layer, which used PLCs and HMIs in order to analyze and process data, enabling real-time decision-making; (c) a communication layer, implementing TCP/IP to facilitate a secure and seamless data exchange between the physical and virtual environments; (d) an application layer, comprising Unity 3D and the HMI, enabling the visualization, interaction, and remote monitoring of industrial processes; (e) an cloud integration layer, responsible for ensuring data storage, predictive maintenance, and analytics-based decision-making by leveraging cloud computing technologies. This architecture enhanced the flexibility and efficiency of the Digital Twin, enabling real-time monitoring, predictive analytics, and remote supervision of industrial processes.

**Figure 1:**
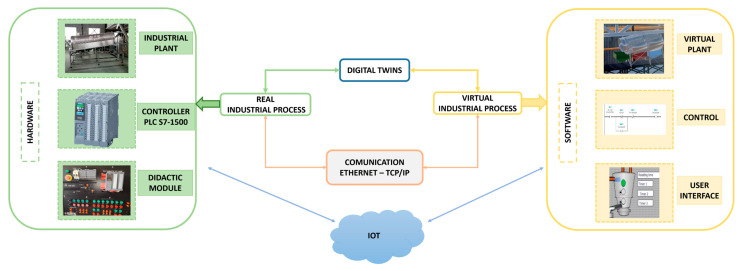
Digital Twin design scheme.

Figure 1 shows the scheme for implementing a Digital Twin in cocoa production. The development of the work consisted of a virtual part (software) and a physical component (hardware). The software consisted of two main stages: (i) the control stage, where the process was programmed in TIA Portal v17 (Simens, Germania) using Ladder language and implementing a sequential control, and (ii) the virtualization stage, where the plant was recreated in a virtual environment, designing a user-friendly Human–Machine Interface (HMI), which made it possible to visualize the process in real time. The hardware consisted of a control panel with a controller based on a PLC S7-1500 with CPU 1511-1 PN. This module facilitated the connection and provides indicators to visualize the process sequence. TCP/IP protocol was used to link the Digital Twin with its respective control. Once programming and virtualization were completed, IoT communication was established by connecting the physical plant elements via the Simatic IOT 2040 module.

## 3. Active Learning Tool Development

### 3.1. Description of the Industrial Process

**Figure 2:**
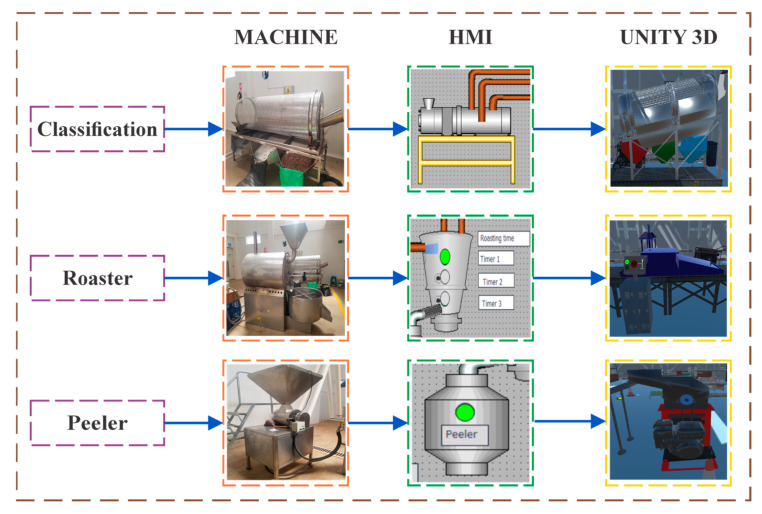
Stages of the industrial process of cocoa production.

This section details the main stages of the industrial process considered in the development of an active learning tool, designed for eighth-level engineering students of mechatronics. The focus was on an industrial process of cocoa production, which represents an integral transformation of the raw material into essential ingredients for the production of chocolate and other derived products. This process is structured in a series of key stages that ensure the quality and consistency of the final product. This article specifically addressed the stages of grading, roasting, and peeling, which were considered fundamental to ensure high quality cocoa, as shown in Figure 2.

Figure 2 details the three main stages of the cocoa production process, starting with (i) sorting of raw material, which requires maximum control in the selection of cocoa, which is considered a critical action in the production process. This is considered a critical action in the production process, since it must ensure the uniformity and quality of the final product and prevent failures in later stages. When the cocoa is received at the plant, the raw material is initially cleaned to eliminate impurities, after which it is channeled to the sorting machines. These machines separate the beans according to size using a system of adjustable brushes, allowing the beans to be categorized into large, medium, and small, as illustrated in Figure 2 [20]. This accurate sorting process is essential to ensure that the sorted kernels meet the technical and quality requirements for subsequent stages. According to recent studies, automated sorting can increase the efficiency of the process by up to 30% (González et al., 2020 [1]).

(ii) Cocoa roasting is a key stage that directly influences the flavor and aroma of the final product. This process involves heating the beans in specialized machines at controlled temperatures, generally between 120 °C and 150 °C, for a period from 10 to 45 min, depending on the size of the beans. During roasting, chemical reactions are generated that enhance the aromatic compounds characteristic of cocoa. Proper time and temperature management is essential to avoid over-roasting that could compromise the quality of the product. Studies by [18] have shown that precise temperature control during roasting improves the sensory perception of cocoa by 15%. Once roasting is complete, the beans are rapidly cooled to lock in the developed flavors, ensuring that they are ready for further processing steps, such as grinding and chocolate manufacturing [21]. And finally, the (iii) cocoa peeling stage aims to separate the shells from the cocoa beans, which is achieved by means of a worm screw mechanism. This system uses a rotating helical shaft that generates friction and pressure, causing the shells to break. The grains then pass through a dehulling machine equipped with a centrifugal separation system, which separates the heavy solids from the light ones. This process produces clean, husk-free grains. Studies by [29] have evidenced that the use of state-of-the-art hulling machines reduces grain loss by 8%. The process is finished with a visual inspection, to ensure that the beans are completely free of impurities, which ensures the purity and quality required for the production of cocoa products [22].

### 3.2. Industrial Process Virtualization

The development of the active learning tool required the development of an interactive work environment that faithfully reproduced the operations of a real industrial plant; the work environment must consider, in detail, the operating characteristics of the equipment and instruments, as well as the dimensions and properties of the physical environment in which the process takes place. This includes the structure of the grading, roasting, and peeling machines, as well as the associated transport systems. It also considers the operational indicators of actuators and sensors of the process, which allow providing real-time feedback on the state of the system, facilitating informed and timely decision-making.

In addition, in order to enhance simulation with the goal of evaluating students’ response to hypothetical scenarios, the Digital Twin system integrated a predictive analytics model that allowed users to simulate various operating conditions beyond standard procedures. Within the Unity 3D environment, process variables such as temperature, pressure, and operating speed could be adjusted to examine their impact on system performance. Also, failure scenarios—including sensor failures, actuator failures, and emergency shutdowns—were incorporated to analyze the effectiveness of different control strategies and optimize response protocols. These simulations were validated using real-time information from the PLC and HMI, ensuring that the system dynamically adapted to changing conditions. This approach provided a risk-free environment to test and optimize industrial operations, enhancing students’ ability to make informed decisions in real-world applications.

**Figure 3:**
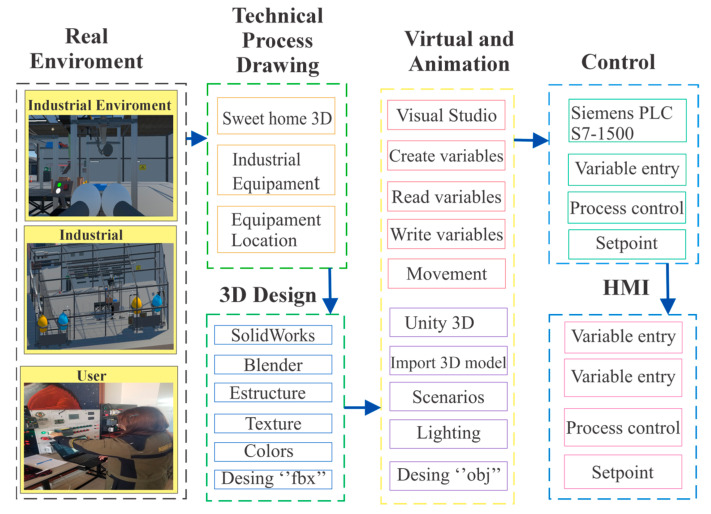
Virtualization phase of the learning tool.

After these characteristics were compiled, the virtual environment was developed using a systematic approach divided into five phases, detailed in Figure 3:

1. Process Technical Drawing Phase: A preliminary sketch of the equipment was created using Sweet Home 3D software (version 7.4), which allowed the initial layout and sizing of the industrial components to be developed.
2. 3D Design Phase: Three-dimensional models of the equipment were built and assembled in SolidWorks 2024. It was crucial that the machines were not designed as single solids but as assemblies of major components that could move independently. The files obtained were exported in STL format and refined in Blender, where colors, textures, and physical attributes were added. Finally, the models were exported as FBX files to be imported into Unity.
3. Virtualization and Animation Phase: This phase began with the definition of the objects and the verification of the system requirements. The development environment was prepared, the 3D models were created and textured, and the assets were organized in Unity. Subsequently, the scene elements, such as lighting and cameras, were configured. The rigging of the models enabled their animation, which was managed by Unity’s Mecanim system. The integration of scripts made it possible to manage the states of the animations and to control the logic of the system.
4. Interactive Simulation Phase: The communication between the virtual environment and a Programmable Logic Controller (PLC) was established through a specific protocol. This ensured that the actions and responses of the virtual system accurately reflected the behavior of the physical system. The simulation also included synchronization between actuators and sensors, allowing a realistic and efficient representation of the process.
5. HMI Integration Phase: A Human–Machine Interface (HMI) was incorporated to facilitate the interaction between the operators and the virtual system. The HMI allowed real-time monitoring and adjustment of process parameters, providing manual control over PLC actions and visualization of operational data.

Finally, a comprehensive optimization of the virtual environment and models was carried out to improve performance, followed by extensive testing to identify and troubleshoot potential problems. In the virtualization environment, it is essential to accurately represent the movements and actions of the actuators, synchronized with the signals detected by the sensors during the process. This level of detail ensures that virtual simulations not only faithfully reflect the behavior of the physical system but are also effective tools for teaching and active learning in engineering.

### 3.3. Human–Machine Interface Development

**Figure 4:**
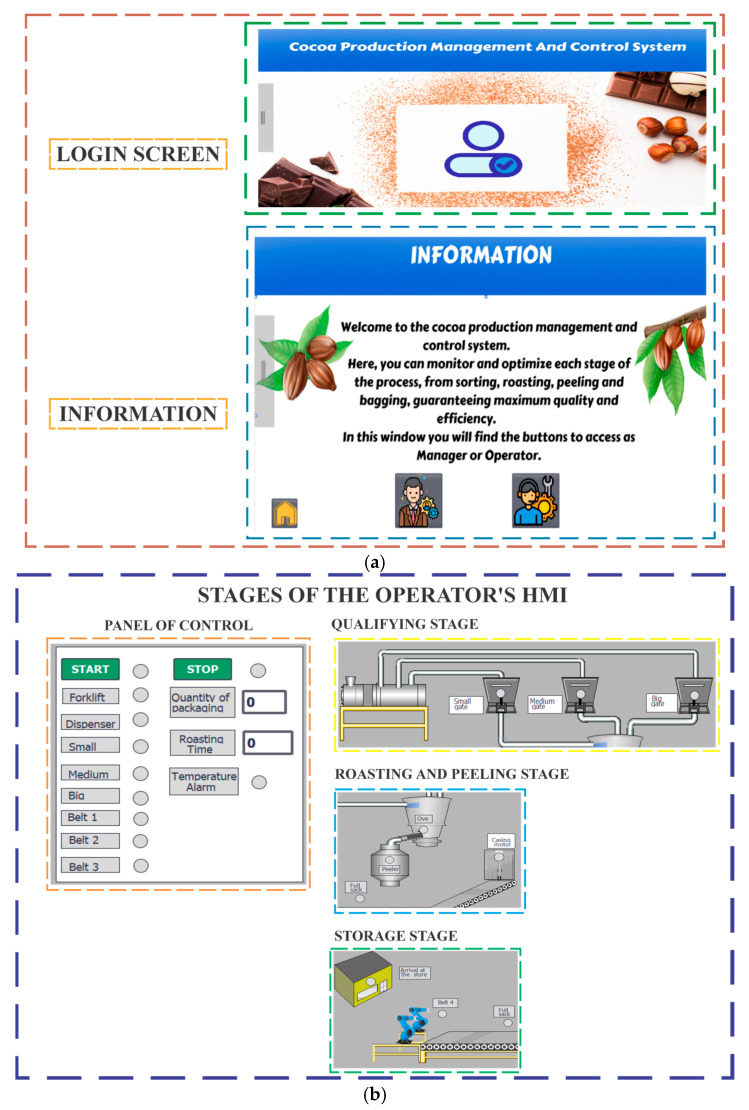
Human–Machine Interface screen. (**a**) system input and information screens; (**b**) process operation screen.

For the development of the Human–Machine Interface (HMI), WinCC v17 software was used, a robust and widely used tool in industrial automation systems that allows the creation of intuitive and efficient interfaces. This system collected real-time data from sensors and devices distributed throughout the industrial plant, facilitating supervision, control, and direct interaction with production processes. In the context of the cocoa sorting, roasting, and peeling process, WinCC was used to develop a user-friendly interface that optimized system management and monitoring, as shown in Figure 4.

Figure 4 shows the HMI configuration. The system started with the design of several screens, each oriented to meet the specific needs of the users involved. The first screen focused on user access management, ensuring an adequate level of security and control. The second screen provided general system information, offering a clear and detailed overview of the operating parameters. And finally, the system operation screen provided information and privileges according to the type of user who accessed the system. That is, if the access was with a manager password, detailed information of the cocoa treatment process was presented in a read-only format, guaranteeing the integrity of the data by allowing the editing of entries. While if logged in as an operator, the interface offered essential interactive elements, such as start and stop buttons, settings to set the start-up time of the grading machine motor, and options to select the number of bags of cocoa to be produced according to size. This integration ensured a balance between strategic oversight and operational control, promoting both process efficiency and an active learning experience for users.

The communication between the HMI developed in WinCC and the automation devices was established using the S7 Communication protocol, which is compatible with Siemens S7 series PLCs. This protocol allowed efficient data transmission between HMI systems and controllers, ensuring accurate and reliable synchronization. In the design of the HMI, the process sequence was graphically recreated, considering the location of the machines, sensors, actuators, and the necessary information to clearly identify each stage of the cocoa processing. This visual and organized approach not only facilitated supervision but also allowed the operator to identify possible system failures and make quick decisions to perform the necessary maintenance and resume the process immediately.

A prominent aspect of the developed HMI is its ability to foster active and immersive learning. By providing a dynamic and easy-to-understand interface, the system allows users to interact directly with the virtual environment, promoting the acquisition and consolidation of technical knowledge. This is especially relevant in educational and training environments, where operators and students can gain a deeper understanding of how industrial processes work. Interaction with the HMI not only allows them to develop critical skills for real-time management and problem solving but also enriches their learning experience, preparing them to face real challenges in the field of industrial automation.

### 3.4. IoT Configuration

**Figure 5:**
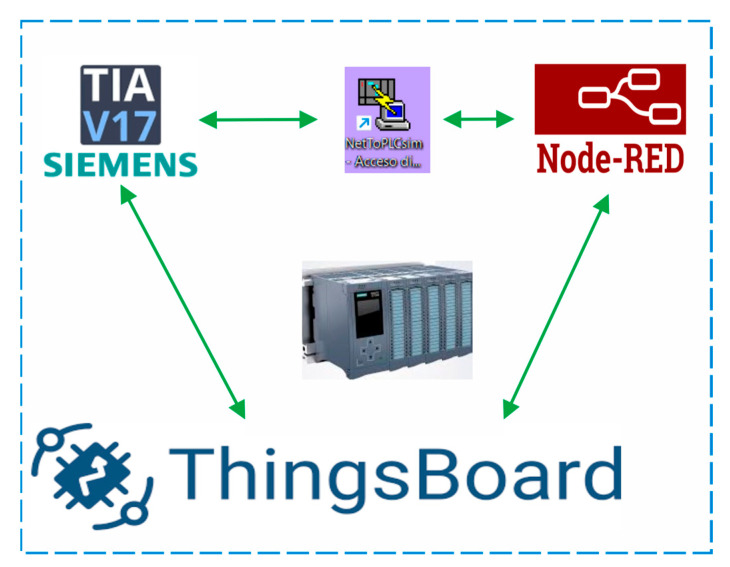
Node-RED and ThingsBoard communication scheme.

The implementation of Internet of Things (IoT) technologies in the virtualization of a cocoa processing plant allows the development of an advanced digital model that optimizes the real-time control and monitoring of the grading, roasting, and peeling processes. This approach not only improves operational efficiency and final product quality but also generates critical data for decision-making, resulting in a more intelligent and adaptive production environment. The IoT system architecture was designed to integrate essential technological tools and devices for process automation, as shown in Figure 5.

In this structure, TIA Portal V17 was used to program and configure the PLC that coordinates the plant’s main equipment, such as the grader, roaster, and peeler, ensuring synchronized and efficient operations. NetToPLCSim (version 1.2.5) played a crucial role, since it allowed simulation and validation of the control logic before its implementation in the real environment, thus minimizing risks and downtime in production. Meanwhile, Node-RED (version 3.1) acted as a middleware that processed and integrated real-time data coming from the PLC. In other words, it automatically regulated parameters such as roasting machine temperature, dynamically adapting to process conditions and ensuring uniform, high-quality roasting. These automatic adjustments contributed significantly to maintaining the consistency of the final product. And finally, ThingsBoard (version 3.8.1) centralized and visualized all the information generated by the control systems and sensors distributed throughout the plant through an intuitive dashboard, allowing operators to monitor key indicators, such as equipment performance and process conditions; identify areas for improvement; and make informed decisions to maintain optimal production. In addition, this dashboard provided alerts and historical analysis that facilitated failure prediction and preventive maintenance, strengthening plant resilience.

## 4. Experimentation of the Tool Designed

This section presents the results of the experimental tests carried out with the developed learning tool, which aims to complement the teaching–learning process through an active learning approach. This tool has been designed for engineering students to interact with virtualized industrial environments, developed with a high level of realism to facilitate immersion and interaction with control systems. Through this environment, students can directly interact with the behavior of actuators and sensors in response to signals emitted by themselves, allowing them to experiment in a safe and controlled space without compromising real physical equipment. The experimental design focused on evaluating the effectiveness of the Digital Twin as a teaching resource, considering key metrics such as the level of understanding of the process, the precision in manipulating the system, and the ability of students to diagnose and solve problems in real time. To this end, a work environment was implemented where students were able to operate an industrial process through a physical PLC, which established communication with the virtual environment using industrial automation protocols. This approach allowed realistic operating conditions to be replicated, ensuring that actions performed in the Digital Twin were faithfully reflected in the process simulation.

### 4.1. User Interaction—Digital Twin

The effectiveness of the Digital Twin as a learning tool depends on the smooth interaction of the user with the developed system. To evaluate system performance, key metrics were analyzed, such as the accuracy of PLC parameter settings, response time to unexpected events, and the ability to correlate process signals with the behavior of the virtualized system. In addition, performance indicators such as user input recognition accuracy, synchronization time between HMI and PLC logic, and error correction efficiency were evaluated to measure the effectiveness of the tool in improving students’ understanding of industrial automation.

**Figure 6:**
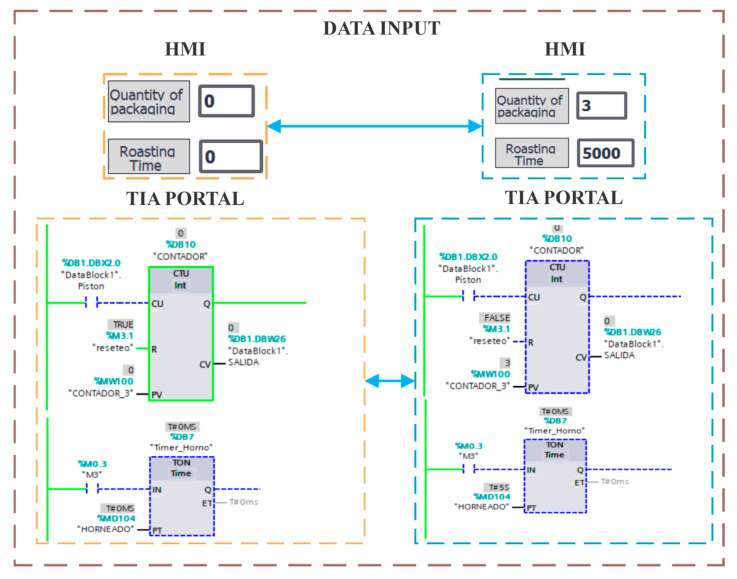
Real-time data entry from TIA Portal V17.

The experimental setup was conducted in an industrial automation lab, where 16 eighth-semester mechatronics students programmed and parameterized a PLC using TIA Portal V17. The students integrated sensor and actuator signals, allowing real-time visualization of the system responses within a virtual environment developed in Unity. The interaction between the PLC, the Digital Twin, and the HMI (WinCC) was essential to ensure a bidirectional exchange of data and commands. Figure 6 illustrates a critical aspect of system operation, showing the transition from initial user input to real-time updates within the HMI and PLC logic. The left section of the diagram represents the initial state, in which no user-defined parameters have been entered, while the right section shows the updated system response following data entry. Specifically, the Packaging Quantity and Roasting Time parameters were modified through the HMI, which caused a real-time update within the PLC logic, as shown in TIA Portal V17.

The lower sections of the diagram show the internal PLC logic, with a counter (CTU) controlling the number of operations executed and a timer (TON) regulating the toasting time. These control elements are crucial to synchronize the physical and virtual components of the system, ensuring that every interaction on the HMI accurately reflects the process variables in the Digital Twin.

**Figure 7:**
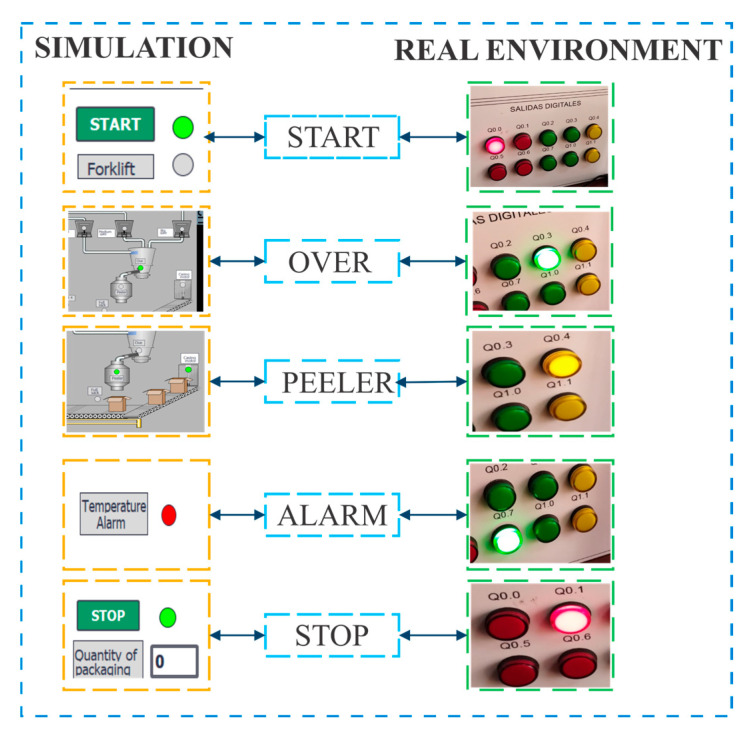
Connection test between the Digital Twin and the real system via the HMI.

To further validate the accuracy of the Digital Twin, a connection test was carried out between the real system and the HMI, as shown in Figure 7. This test allowed comparing the behavior of the virtualized system with a real industrial automation environment, evaluating signal synchronization and actuator response.

During the test, five key events in the automated process were analyzed: system activation (START), operation status (OVER), peeler operation (PEELER), activation of a temperature alarm (ALARM), and process termination (STOP). It was verified that each command entered in the HMI generated an immediate and consistent response in the physical system, observing the corresponding activation in the PLC digital output modules. The results showed minimal discrepancies in response times, confirming that the synchronization between HMI inputs and PLC logic updates remained within an acceptable range. This behavior demonstrates the robustness and fidelity of the system integration, ensuring that the Digital Twin highly accurately reflects the actual process operating conditions.

**Figure 8:**
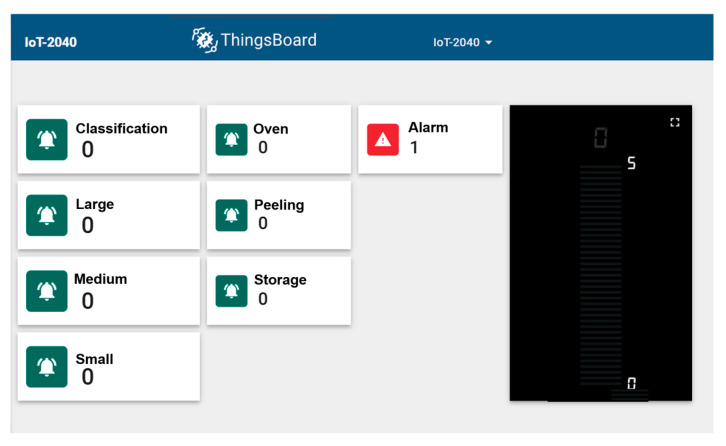
Validation of Digital Twin synchronization with the real system via HMI.

In addition to the HMI-based interaction, the system includes a real-time visualization application implemented on the ThingsBoard platform. This application provides an interactive dashboard that displays critical process stages, including classification, roasting, and peeling, as well as the type of cocoa bean being processed. The visualization system was fully synchronized with the developed Digital Twin, offering real-time monitoring of process variables. Moreover, if the temperature deviated from optimal conditions, the system triggered an alarm notification, alerting the user and enabling prompt corrective action. This additional layer of interaction significantly enhanced the situational awareness of users, facilitating real-time decision-making and improving process control efficiency (see Figure 8).

Finally, a qualitative assessment was conducted through student feedback, highlighting the accuracy and realism of the Digital Twin as a learning tool. A significant improvement was observed in students’ ability to program PLCs, interpret sensor and actuator signals, and diagnose process deviations. The interactive nature of the system reinforced theoretical concepts while enabling students to develop practical skills in a safe and controlled environment.

### 4.2. Virtualized Environment of the Digital Twins

**Figure 9:**
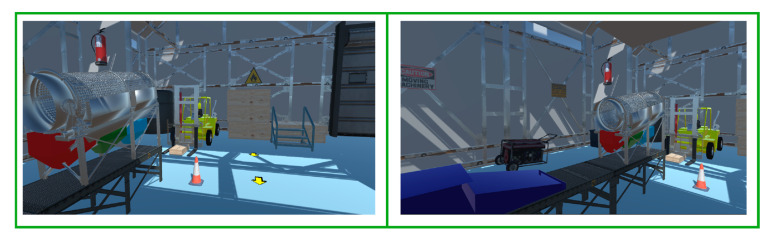
Immersive visualization and interaction in the Digital Twin environment.

In the development of this work, a virtualized environment was implemented using Unity for the digital representation of the industrial process, which improved teaching and learning through immersion and interaction in a safe environment. The simulation integrates high-fidelity 3D models, real-time physics and interactive interfaces that replicate the behavior of industrial systems. Immersion is achieved through detailed visualization of components, realistic lighting, and simulation of operating conditions, while interaction is facilitated through navigation control, manipulation of system elements, and real-time feedback (see Figure 9).

**Figure 10:**
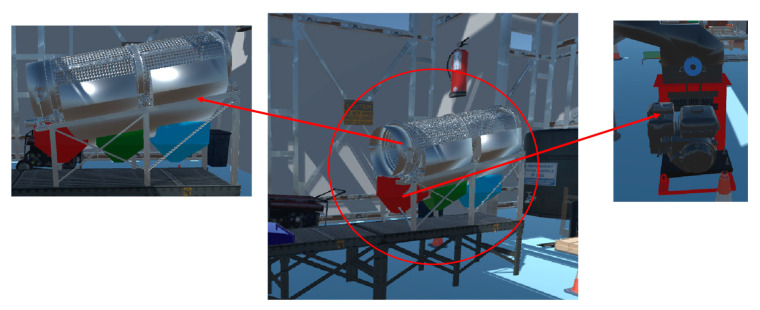
Virtualization of the cocoa processing machine in the Digital Twin environment.

In addition, a high level of realism was incorporated in the simulation of the cocoa preparation machine, as shown in Figure 10. Virtualization of the key stages of the process, including grading, roasting, and peeling, has been developed with detailed and textured models to improve visual fidelity and make this tool a more effective learning resource. The virtualized machine animation has been carefully designed to represent the actual operation of the equipment. In the sorting stage, the rotary motion of the machine was observed, allowing users to understand its operating mechanics. During roasting, the temperature value of the system was displayed in real time and dynamically adjusted according to the type of bean selected, ensuring optimal conditions and preventing the product from burning. Finally, in the peeling phase, the movement of the roller in charge of removing the husk from the bean was accurately simulated, after which the product was transported by a conveyor belt, completing the processing cycle.

This virtual environment not only allows detailed observation of the machine’s operation but also encourages active user interaction. The ability to manipulate system variables and visualize machine responses in real time contributes significantly to the understanding of the industrial process. In addition, virtualization provides a safe learning environment, eliminating risks associated with the operation of real machinery and protecting both students and industrial equipment. From an educational perspective, this application strengthens active learning by allowing students to experiment with system variables, analyze their effects, and optimize parameters without negative consequences. The combination of immersion, interaction, and accurate simulation transforms this environment into a valuable tool for engineering education, providing a close-to-reality experience and enhancing the development of technical and analytical skills essential in industry.

**Figure 11:**
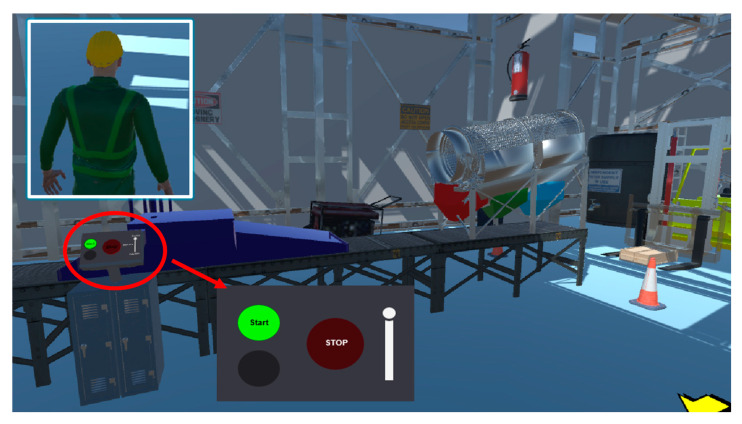
Bidirectional control and user immersion through Digital Twin interaction.

Finally, control of the system was performed bilaterally, allowing both remote monitoring and direct interaction with the virtualized environment. The inclusion of an avatar within the system contributed significantly to the immersion of the user, providing a visual representation of his presence in the simulated environment. This avatar not only enhanced the sense of presence in the simulation but also facilitated interaction with the various components of the system, guiding the user through the industrial processes represented in the simulation (see Figure 11). Because of this approach, the Digital Twin platform offers a more intuitive and immersive learning experience, enabling a deeper understanding of industrial processes and optimization of operating parameters in a safe and controlled environment.

### 4.3. Digital Twin Impact Assessment

This subsection evaluates the impact of the Digital Twin on active learning applied to the industrial process of cocoa, for which experimental tests were carried out with a group of 16 eighthsemester students of mechatronics. The study group consisted of 5 women and 11 men, who presented a homogeneous level of technical and theoretical skills. All the participants had experience in the design of industrial process control systems and in the use of PLCs through didactic modules.

For the evaluation, an experimental design based on two phases was implemented: (i) Pre-test with traditional methodology: initially, the students performed a practical test using only conventional didactic modules for the programming and control of industrial processes. This was proceeded with (ii) intervention with the Digital Twin and post-test: one week later, the same students were exposed to a learning environment based on the Digital Twin, developed in an active learning laboratory. We proceeded to apply a post-test in order to compare the results obtained in both methodologies and measure the effectiveness of the digital approach in the acquisition of knowledge and skills.

The results collected from the experimental tests are presented in Table 1, which summarizes the students’ performance in both phases. Three key indicators were analyzed: accuracy in responses, time taken to complete the test, and error rate. The data show a significant improvement after the implementation of the Digital Twin, with an increase in accuracy from an average of 68.4% in the pre-test to 89.2% in the post-test. Similarly, the average time required to complete the test decreased from 18.2 min to 12.5 min, reflecting a greater efficiency in problem-solving. Additionally, the percentage of errors was notably reduced from 21.6% to 7.8%, demonstrating an improvement in the students’ ability to apply industrial automation concepts effectively.

These results confirm the positive impact of the Digital Twin in active learning, as students demonstrated not only a better understanding of industrial automation concepts but also improved problem-solving efficiency. The reduction in errors and execution time suggests that the integration of virtualized environments enhances the assimilation of knowledge, providing a safer and more interactive learning experience.

**Figure 12:**
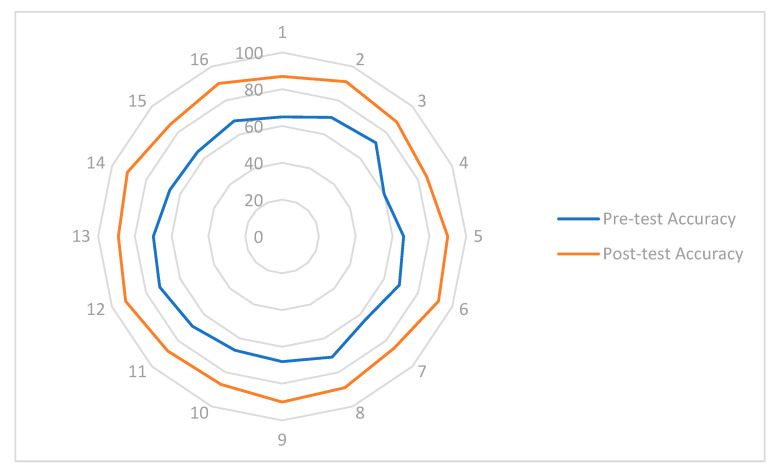
Accuracy test analysis of the developed system.

The comparison of system manipulation accuracy between the pre-test and post-test was analyzed, as shown in Figure 12. This analysis highlights the positive impact of Digital Twin technology on the active learning process. It was observed that accuracy in the manipulation and control of the cocoa production system using Digital Twin technology was significantly higher compared to the traditional methodology. In the pre-test, where students worked exclusively with conventional teaching modules, the average accuracy was approximately 67.63%, revealing certain difficulties in programming and system configuration. In contrast, following the intervention with the Digital Twin and the completion of the post-test, the average accuracy increased to 88.81%, reflecting a 21.18% improvement in students’ ability to understand and operate the system.

This increase in accuracy is attributed to the real-time interaction students experienced with the Digital Twin, enabling them to visualize and adjust parameters in a safe and dynamic environment. Additionally, the digital model facilitated the correlation between process signals and system responses, reinforcing conceptual understanding and the development of practical skills essential for industrial process control.

**Figure 13:**
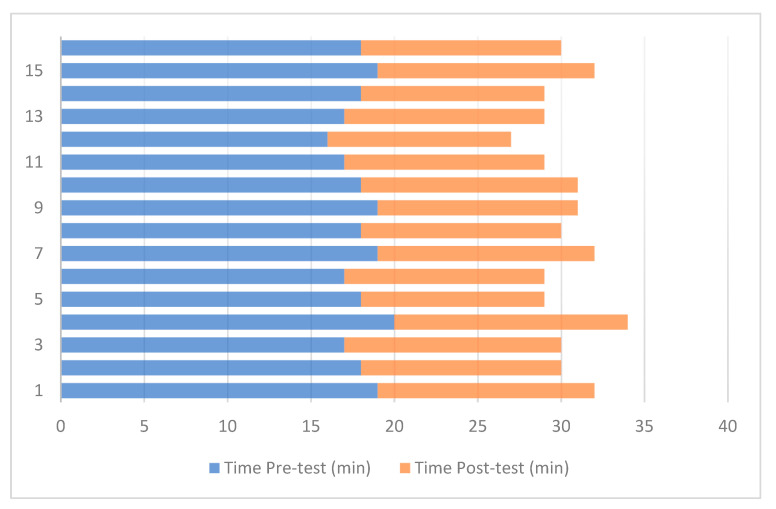
Analysis of system handling time.

Figure 13 presents a comparison of the time students took to manipulate the system using the traditional method versus the active learning tool based on Digital Twins. A significant reduction in the time required to complete tasks was observed following the intervention with the Digital Twin. During the pre-test, where students worked with conventional teaching modules, the average time to program and control the system was approximately 18 min. In contrast, in the post-test, where students interacted with the Digital Twin, the average time was reduced to 12 min, representing a 6 min decrease in execution time.

This reduction in manipulation time is attributed to the real-time interactive visualization offered by the Digital Twin, enabling students to identify and correct errors more efficiently. Furthermore, the digital environment provided a clearer understanding of the relationships between process parameters and their impact on the system, enhancing decision-making and improving students’ ability to manipulate the cocoa production system.

### 4.4. Evaluation of the System Performance

In order to evaluate the effectiveness of the Digital Twin-based learning tool developed, an evaluation focused on three key indicators: system adaptation time, decision-making accuracy, and level of knowledge transfer. These indicators provide further insight into how students interact with the platform, apply the acquired concepts, and transfer their knowledge to practical industrial scenarios.

A. System Adaptation Time

To assess how students adapt to handling and controlling the Digital Twin environment, their adaptation time is evaluated. This is measured from the initial interaction with the system until the student is able to complete the assigned tasks independently, without external assistance. Adaptation times were recorded for each student and categorized into three levels: Fast (≤10 min), Moderate (10–20 min), and Slow (≥20 min) (See Table 2).

The classification of system adaptation time into three levels is based on established benchmarks in the field of human–computer interaction and active learning in industrial automation environments. Studies on learning curves in Human–Machine Interfaces (HMI) suggest that an adaptation period of up to 10 min is indicative of an intuitive and user-friendly interface, while times between 10 and 20 min reflect a moderate learning curve where users require some initial guidance but eventually achieve operational fluency. Adaptation times exceeding 20 min suggest a steeper learning curve, indicating that the interface or control logic may require further simplification or additional instructional support. To validate this classification, a preliminary usability evaluation was performed in which students were asked to navigate the interface and complete a basic sequence of tasks without prior instruction. The adaptation thresholds were determined based on the observed time intervals where students demonstrated autonomous system operation without external assistance. This categorization allows for an objective measurement of usability and ease of interaction, providing valuable insights into the effectiveness of the Digital Twin as an educational tool.

B. Decision-Making Accuracy

Students were evaluated on the basis of their accuracy in decision-making with respect to the work they perform in the different simulated industrial scenarios. For this purpose, five operational challenges were presented to each student. Among them, sensor failures, actuator failures, and emergency stop situations were included. Accuracy was calculated as the percentage of correct actions performed in response to each scenario. Table 3 shows the accuracy scores, which indicate that the students effectively applied their theoretical knowledge to solve practical challenges, reinforcing the role of the Digital Twins in decision-making training.

The evaluation of decision-making accuracy was based on five operational challenges designed to replicate real-world industrial scenarios. These included sensor failures, actuator malfunctions, emergency shutdowns, process optimization tasks, and system overload responses. Each scenario required students to diagnose issues, apply corrective actions, and optimize system performance. Performance was assessed using three criteria: accuracy in identifying the fault, effectiveness of the corrective action, and response time. The classification of responses followed a structured rubric to ensure objective evaluation and reproducibility. By defining these challenges based on common failure modes in industrial automation, the assessment provides a standardized framework to measure student proficiency in problem-solving and decision-making under realistic conditions.

C. Knowledge Transfer Level

Finally, to measure the transfer of knowledge, we proceeded to a post-test in which the students had to apply the concepts learned through the Digital Twin environment to a real industrial control problem. The evaluation was based on their ability to identify the correct control parameters, propose an optimized operating strategy, and implement system changes with minimal errors. The results were classified into three performance levels: high, medium, and low transferability. To ensure an objective assessment of knowledge transfer, a structured rubric was implemented to classify student performance into three levels: high (≥80%), medium (60–79%), and low (<60%). The classification was based on three key aspects: (1) accurate identification of control parameters, (2) effectiveness in optimizing operational strategies, and (3) correct implementation of system adjustments. The scoring methodology was validated through expert review, ensuring alignment with industrial training standards. This structured approach reduces subjectivity and enhances the reliability of the evaluation, providing a clear measure of how well students can apply the concepts learned in the Digital Twin environment to real industrial scenarios.

As shown in Table 4, most students demonstrated a high or medium level of knowledge transferability, confirming that the Digital Twin-based system improves the ability to apply learned concepts in practical environments.

## 5. Conclusions

The active learning tool developed based on Digital Twin technology has been designed to provide students with a virtualized environment simulating the industrial cocoa process. This interactive and immersive teaching approach not only enhances the understanding of concepts but also ensures a safe environment where students can control the system and observe its responses in real time. Additionally, the tool respects the actual physical characteristics of the actuators and sensors that make up the process, offering an educational experience closer to industrial reality. To assess its impact, a comparison was made with the traditional method of teaching engineering, which combines theoretical classes with practical activities using teaching modules. While Digital Twin-based teaching has proven effective in conveying basic concepts, the integration of the digital tool adds an extra layer of interaction and immersion. During its use, students can manipulate the system and observe, in real time, changes in the equipment and instruments, which facilitates a deeper understanding of the dynamics of the industrial process.

The evaluation results, obtained from 16 students, demonstrate a significant improvement in system manipulation accuracy, along with a 38% reduction in task execution time compared to traditional methods. These findings indicate that the tool plays a crucial role in enhancing practical skills and decision-making abilities in industrial process control. However, it is essential to consider this tool as a complementary resource rather than a substitute for theoretical and practical instruction provided by educators. The integration of both methodologies ensures a more comprehensive learning experience, equipping students with the necessary knowledge and skills to tackle real-world industrial challenges effectively. Furthermore, the evaluation results underscore the effectiveness of the Digital Twin platform in industrial automation training. The relatively fast adaptation time suggests that the system interface is well designed for ease of use, allowing students to quickly become proficient. The high decision-making accuracy observed in simulated scenarios confirms that students developed strong analytical and problem-solving skills, which are critical in industrial environments. Lastly, the high knowledge transfer rates indicate that the skills acquired through this training extended beyond the simulated environment, reinforcing their applicability in real-world settings and aligning with the educational objectives of Industry 4.0.

In the future, we are considering expanding the study with a larger number of participants to obtain more representative results and evaluate its impact on different levels of training. In addition, the integration of technologies such as augmented reality and artificial intelligence will optimize the Digital Twin model, improving the accuracy of the tool and its adaptability to different industrial processes. These improvements will not only ensure more dynamic and effective learning but will also reinforce student training, aligning it with the changing demands of Industry 4.0. Beyond its educational scope, the implementation of Digital Twins holds significant potential for industrial applications. The ability to create accurate virtual replicas of physical systems enables real-time monitoring, predictive maintenance, and process optimization in a variety of industries. For example, in cocoa processing and other food industries, a Digital Twin can simulate production scenarios, analyze equipment performance, and predict maintenance needs, reducing downtime and increasing operational efficiency. This work can be extended to broader industrial applications, such as automated quality control, supply chain optimization, and energy efficiency management, bridging the gap between academia and industry.

## Figures and Tables

**Table 1:** Student performance in pre-test and post-test.

Student	Pre-Test Accuracy	Time Pre-Test (min)	Pre-Test Errors (%)	Post-Test Accuracy	Time Post-Test (min)	Post-Test Errors (%)
1	65	19	24	87	13	9
2	70	18	21	91	12	6
3	72	17	19	88	13	8
4	60	20	26	85	14	11
5	66	18	22	90	11	7
6	69	17	20	92	12	5
7	64	19	25	86	13	9
8	71	18	18	89	12	6
9	68	19	21	90	12	7
10	67	18	23	87	13	8
11	69	17	20	88	12	6
12	72	16	17	92	11	5
13	70	17	19	89	12	6
14	66	18	22	91	11	5
15	65	19	23	86	13	9
16	68	18	20	90	12	6

**Table 2:** Student adaptation time.

Adaptation Level	Number of Students	Percentage (%)
Fast (≤10 min)	7	43.75%
Moderate (10–20 min)	6	37.5%
Slow (≥20 min)	3	18.75%

**Table 3:** Accuracy score for different scenarios.

Scenario Type	Average Accuracy (%)
Sensor Failure	85.2
Actuator Failure	78.6
Emergency Shutdown	92.4
Process Optimization	74.3
System Overload	81.5

**Table 4:** Knowledge transfer levels.

Knowledge Transfer Level	Number of Students	Percentage (%)
High (≥80%)	9	56.25%
Medium (60–80%)	5	31.25%
Low (<60%)	2	12.5%

## Data Availability

The data presented in this study can be consulted by mailing the author.
